# Effects of high-quality nursing care on psychological outcomes in patients with chronic heart failure

**DOI:** 10.1097/MD.0000000000017351

**Published:** 2019-10-11

**Authors:** Xiao-Qing Li

**Affiliations:** Department of Infection Control, The Fourth People's Hospital of Shaanxi, Xi’an, China.

**Keywords:** chronic heart failure, effect, high-quality nursing care, psychological outcome, randomized controlled trial

## Abstract

**Background:**

This study will assess the effects of high quality nursing care (HQNC) on psychological outcomes (PCO) in patients with chronic heart failure (CHF).

**Methods:**

We will carry out a through search in 7 databases: PUBMED, EMBASE, Cochrane Library, Web of Science, Chinese Biomedical Literature Database, WANGFANG, and China National Knowledge Infrastructure. Eligibility criteria will be randomized controlled trials on assessing effects of HQNC on PCO in patients with CHF. Cochrane risk of bias evaluation will be utilized for methodological quality.

**Results:**

This proposed study will summarize a rational synthesis of current evidence for HQNC on PCO in patients with CHF.

**Conclusion:**

The results of this study will provide convinced evidence for judging the effects of HQNC on PCO in patients with CHF.

## 1 Introduction

Chronic heart failure (CHF) has become a global epidemic disorder, and it impacts about 26 million populations around the world.^[1–4]^ Such disorder often significantly affects quality of life and brings a tremendous burden for both patients’ family and the society.^[5–8]^ It has been estimated that its prevalence was 0.9%, and the mortality was 19.5% annually in China.^[9]^ In addition, the CHF motility was 17.4% in Europe,^[10]^ and 31.4% in United States each year.^[11]^ Previous studies found significant associations of psychological outcomes (PCO) with CHF.^[12–17]^ Thus, it is very important to manage PCO in patients with CHF in clinical practice.

Several clinical studies have reported that high-quality nursing care (HQNC) has been utilized to manage PCO in patients with CHF.^[18–22]^ However, no study has systematically assessed the effects and safety of HQNC on PCO in patients with CHF, and its effects are still inconclusive. Therefore, the present study will investigate the effects and safety of HQNC on PCO in patients with CHF.

## 2 Methods

### 2.1 Study registration

This study protocol has been registered on PROSPERO (CRD42019140638). It has been reported abiding to the Preferred Reporting Items for Systematic Reviews and Meta-analyses Protocols statement guidelines.

### 2.2 Eligibility criteria for study selection

#### 2.2.1 Types of studies

All available randomized controlled trials (RCTs) about the use of HQNC on PCO in patients with CHF will be included. Other studies such as case reports, case series, noncontrolled studies, and non-RCTs will be excluded.

#### 2.2.2 Types of participants

We will include studies on patients that have been diagnosed as PCO in patients with CHF without any restrictions of age, sex, and ethnicity.

#### 2.2.3 Type of interventions

The experimental intervention must be HQNC for PCO in patients with CHF.

The control intervention can be any therapies, except HQNC.

#### 2.2.4 Type of outcomes

The primary outcomes are depression and anxiety. They can be measured by Hamilton Depression Rating Scale or Hamilton Anxiety Rating Scale or any other tools.

The secondary outcomes comprise of pain intensity, health-related quality of life, and adverse events. Pain intensity can be assessed by visual analog scale or other scales. Health-related quality of life can be evaluated by 36-Item Short Form Survey, or any other tools. Additionally, any expected or unexpected adverse events will also be measured.

### 2.3 Strategy of literature searches

Relevant studies will be independently and comprehensively searched in PUBMED, EMBASE, Cochrane Library, Web of Science, Chinese Biomedical Literature Database, WANGFANG, and China National Knowledge Infrastructure. The searched items will be utilized as follows: HQNC, PCO, and CHF. The detailed search strategies in Cochrane Library are exerted in Table 1, and will be adapted similarly in other databases.

**Table 1:**
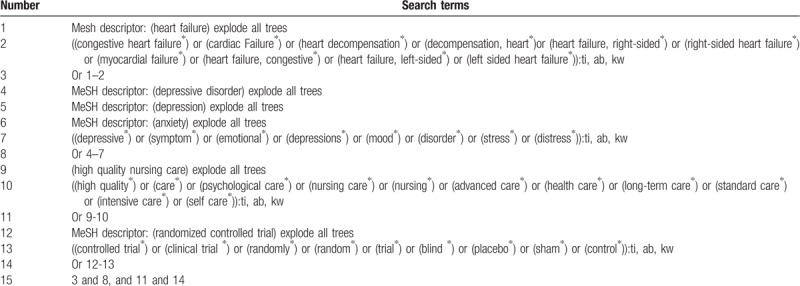
Search strategy used in Cochrane Library database.

Relevant reviews will also be searched. In addition, we will also filter relevant journals and magazines to identify literature to avoid missing any other potential studies.

### 2.4 Data collection

#### 2.4.1 Study selection

**Figure 1:**
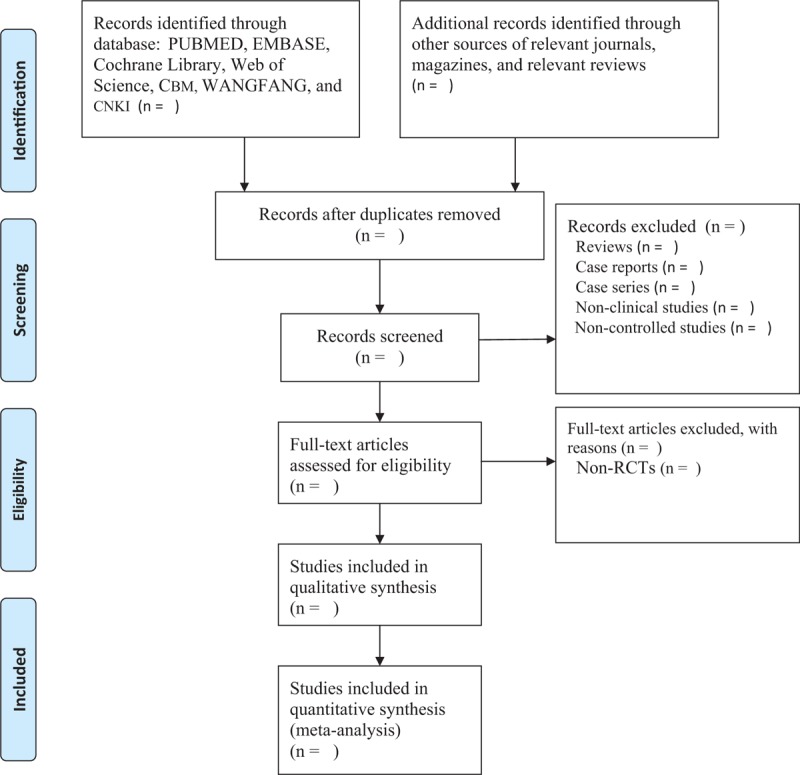
Process of study selection.

Two authors will independently import relevant studies obtained from all searched records into Endnote X7. After excluding duplications, the same 2 authors will independently assess the titles and abstracts of the searched trials and remove unqualified records. Then, the full text of remaining studies will be read carefully to judge if they meet final eligibility criteria. Any differences generated between 2 authors will be solved via discussion with the help of another author. The study selection procedure is showed in a flow chart in Fig. 1.

#### 2.4.2 Data extraction

Data will be independently collected from the eligible studies by 2 authors. Any divergences on data collection between 2 authors will be judged by a third author through discussion. The following information will be collected: title, authors, year of publication, sample size, patient characteristics, study setting, study methods, treatment details, comparators, outcome measurements, and follow-up details. We will contact primary authors for the above mentioned data if they are unclear, ambiguous, or insufficient.

### 2.5 Assessment of risk of bias

Methodological quality of all eligible studies will be assessed according to the Cochrane risk of bias by 2 independent authors. Any discrepancies between 2 authors will be solved by discussion with a third author if it is necessary. The tool includes 7 aspects, and each item will be further divided into 3 levels of low, unclear, and high risk of bias.

### 2.6 Measures of treatment effect

For continuous data, we will apply mean difference or standardized mean difference and 95% confidence intervals (CIs). For dichotomous data, we will use risk ratio and 95% CIs.

### 2.7 Assessment of heterogeneity

We will assess the heterogeneity with the use of *I*^2^ test. *I*^2^ ≤50% may represent reasonable heterogeneity, and a fixed-effect model will be utilized. *I*^2^ >50% may represent significant heterogeneity, and a random-effect model will be applied.

### 2.8 Data synthesis

All analysis will be performed using RevMan 5.3 software. We will select a fixed-effect model or a random-effect model to merge the outcome indicators based on the results of heterogeneity test. If there is reasonable heterogeneity (*I*^2^ ≤50%), a meta-analysis will be carried out. If there is significant heterogeneity or after subgroup analysis (*I*^2^ >50%), we will report a narrative summary instead of data pooled and meta-analysis.

### 2.9 Subgroup analysis

The following subgroup analysis will be explored the potential sources of the heterogeneity in accordance with different study characteristics, treatments, controls, and outcome measurements.

### 2.10 Sensitivity analysis

We will perform sensitivity analysis to assess the robustness of pooled results by taking away low quality studies.

### 2.11 Publication bias

We will carry out funnel plot^[23]^ and Egger regression^[24]^ to analyze the existence of publication bias if more than 10 studies are included.

### 2.12 Ethics and dissemination

We will utilize publicly available data from previous published studies; hence, no ethical approval is needed. We will disseminate the results of this study through publication at a peer-reviewed journal or conference proceedings.

## 3 Discussion

To the best of our knowledge, this study will be the first study to systematically assess the effects of HQNC on PCO in patients with CHF. First, the results of this study will provide objective statistics for further studies on HQNC. Second, the results of this study will provide references for both clinicians and patients in the invention of HQNC on PCO in patients with CHF. Third, the results of this study may provide alternative intervention of PCO in patients with CHF to the policy makers. Therefore, this study may summarize most recent clinical evidence helping clinicians make decisions on clinical practice for the treatment of PCO in patients with CHF.

## Author contributions

**Conceptualization:** Xiao-Qing Li.

**Data curation:** Xiao-Qing Li.

**Formal analysis:** Xiao-Qing Li.

**Investigation:** Xiao-Qing Li.

**Methodology:** Xiao-Qing Li.

**Project administration:** Xiao-Qing Li.

**Resources:** Xiao-Qing Li.

**Software:** Xiao-Qing Li.

**Supervision:** Xiao-Qing Li.

**Validation:** Xiao-Qing Li.

**Visualization:** Xiao-Qing Li.

**Writing – original draft:** Xiao-Qing Li.

**Writing – review & editing:** Xiao-Qing Li.
